# Flickering task–irrelevant distractors induce dilation of target duration depending upon cortical distance

**DOI:** 10.1038/srep32432

**Published:** 2016-08-31

**Authors:** Miku Okajima, Yuko Yotsumoto

**Affiliations:** 1 Department of Life Sciences, The University of Tokyo, Tokyo, Japan

## Abstract

Flickering stimuli are perceived to be longer than stable stimuli. This so-called “flicker-induced time dilation” has been investigated in a number of studies, but the factors critical for this effect remain unclear. We explored the spatial distribution of the flicker effect and examined how the flickering task-irrelevant distractors spatially distant from the target induce time dilation. In two experiments, we demonstrated that flickering distractors dilated the perceived duration of the target stimulus even though the target stimulus itself was stable. In addition, when the distractor duration was much longer than the target duration, a flickering distractor located ipsilateral to the target caused greater time dilation than did a contralateral distractor. Thus the amount of dilation depended on the distance between the cortical areas responsible for the stimulus locations. These findings are consistent with the recent study reporting that modulation of neural oscillators encoding the interval duration could explain flicker-induced time dilation.

Flickering stimuli have been reported to affect the perceived duration of stimulus intervals. For example, after adaptation to a 20-Hz flickering stimulus, the duration of a 10-Hz flickering stimulus is perceived to shorten^1^,^2^. The lateral geniculate nucleus (LGN) has been suggested to contribute to this temporal-compression aftereffect, because the characteristics of this effect, including location specificity^3^ and absence in the case of equiluminant stimuli^4^, are consistent with the response characteristics of the LGN magno cells^2^. As another example of the effect of flickering stimuli, the interval of a stable stimulus following exposure to a flickering adapter is perceived as longer^5^. Ortega *et al*.^6^ showed the orientation and eye-specificity of this temporal-dilation aftereffect and suggested that V1 neurons contribute to it. These examples indicate that several neural areas are involved in time perception and that recruiting different neural areas can result in different kinds of time distortion. Such a complexity has made it difficult to understand the neural mechanism of time perception.

It has also been reported in a number of studies that the duration of the flickering stimulus itself is perceived to be longer than the actual duration^7^,^8^,^9^,^10^,^11^,^12^,^13^,^14^,^15^. Despite the robustness of this “flicker-induced time dilation”, the mechanism contributing to this effect remains unclear. The critical factor for flicker-induced time dilation is highly controversial. Herbst *et al*.^12^ suggested that the subjectively perceived saliency of a stimulus is essential for flicker-induced time dilation. Kanai *et al*.^8^ showed that the dilation size of the perceived duration increases with the increasing temporal frequency of a flicker. However, Kaneko and Murakami^16^ concluded that the speed of a stimulus explains duration dilation better than does the temporal frequency. Recently, Hashimoto and Yotsumoto^14^ suggested that flicker-induced time dilation could be explained by neural entrainment of temporal oscillators.

In this study, we examined which factor is critical for flicker-induced time dilation by exploring the spatial distribution of the flicker effect. In two experiments, participants reproduced the duration of the target stimulus by pressing a button while ignoring the task-irrelevant distractor stimulus. If the target properties such as saliency, temporal frequency, or speed, are critical for flicker-induced time dilation, the flickering distractors would not induce time dilation of the stable target. On the other hand, if neural entrainment is critical, the flickering distractors would induce time dilation.

From studies using electroencephalogram (EEG), it is well known that visual flickering stimuli evoke a steady-state visually evoked potential (SSVEP)^17^,^18^,^19^. SSVEPs have been found at the same frequency as the flicker frequency, and have been localized to the early visual cortical areas^20^,^21^,^22^, which exhibit retinotopic maps^23^. In addition, an SSVEP is evoked by task-irrelevant flickering stimuli^24^,^25^,^26^, and propagate across cortical areas^27^. Notbohm, Kurths, and Herrmann^28^ examined whether the origin of SSVEP is neural entrainment of brain oscillators or superposition of event-related responses. By showing the degree of SSVEP depends on the difference between the flicker frequency and the Individual Alpha Frequency, they demonstrated that visual flickering stimulation results in entrainment of brain oscillators, and that the entrainment is the underlying mechanism of SSVEPs. Therefore, it can be assumed that a flickering task-irrelevant distractor induces neural entrainments, and that the neural entrainments propagate across cortical areas in the retinotopic manner. In fact, sensory stimuli with temporal modulations are broadly accepted as an eligible tool to investigate functions of the neural entrainments^7^,^14^,^28^,^29^,^30^,^31^.

Because neural entrainments propagate across cortical areas^18^,^27^, the effect of the flickering distractor on neural entrainment would depend not on the retinotopic distance but on the cortical distance. Hence, we positioned the distractor in the ipsilateral or contralateral visual hemifield of the target in order to manipulate the cortical distances between the target and the distractors. Even when the retinotopic distances between the target and the distractor were constant, the ipsilateral flickering distractor would have a greater effect of neural entrainment than would the contralateral flickering distractor, because the cortical distance to the target from the ipsilateral distractor would be shorter than that from the contralateral distractor. With simulations based on the computational model, Hashimoto and Yotsumoto^14^ demonstrated that neural entrainment can explain the flicker-induced time dilation: weak neural entrainment induces little time dilation and strong neural entrainment induces large time dilation. Therefore, if neural entrainment is critical for flicker-induced time dilation, the amount of time dilation would depend on the cortical distance between the flickering distractor and the target: the flickering ipsilateral distractor would have larger effect of neural entrainment and induce larger time dilation than the flickering contralateral distractor.

It is possible that flickering distractors would reduce attention to the target and thus alter perceived target properties such as saliency, frequency, or speed. If so, however, the effect of the distractor would be independent of whether the distractor was ipsilateral or contralateral, as a previous study has demonstrated that higher-level attention is not modulated by whether attention is divided across or within the hemifield^22^. In addition, a general arousal level can also be altered by the flickering distractors. Actually, New and Scholl^32^ has demonstrated that the oddball spatially distant from the target induced time dilation and argued that this reflected the increased general arousal because the dilation effect was independent of the oddball position. Therefore, if a general arousal level matters, the time dilation amount would be independent of the flickering distractor position.

Two stimuli—target and distractor—were used in the experiments (Fig. 1). The target stimulus was a white disk and was displayed in either the upper left or upper right visual field. The distractor stimulus was a green disk and was displayed in a location either ipsilateral or contralateral to the target. The stimuli were either stable or flickering at 10 Hz.

Each experiment included four conditions, which were named on the basis of whether the target and the distractor were stable or flickering and whether the distractor was ipsilateral or contralateral (Fig. 2). Under SS condition (Stable target and Stable distractor), a stable target and stable distractor were displayed. Under FS condition (Flickering target and Stable distractor), a flickering target and stable distractor were displayed. Under SFi condition (Stable target and Flickering distractor ipsilateral), a stable target and a flickering distractor ipsilateral to the target were shown. Under SFc condition (Stable target and Flickering distractor contralateral), a stable target and a flickering distractor contralateral to the target were shown.

We conducted two experiments. In Experiment 1, we used a distractor with a duration much longer than the target duration in order to maximize the effect of the flickering distractor (Fig. 3a). In Experiment 2, the distractor duration was almost the same as the target duration in order to probe the effect of the distractor duration (Fig. 3b).

## Results and Discussion

### Experiment 1

In Experiment 1, we examined the effect of a task-irrelevant flickering distractor on the perceived duration of the target. To make the effect of the flickering distractor as large as possible, the distractor duration was much longer than the target duration.

For statistical analyses, we applied Bayesian inference to dissociate the fixed effect of time dilation from the random effect of the individual. By applying the Markov-chain-Monte-Carlo sampling, the fixed effects of each condition were estimated. The estimated effects of the FS, SFi, and SFc conditions are shown in Fig. 4a. These estimated values are the ratios of reproduced durations compared with that under SS condition. The estimated values for FS, SFi, and SFc conditions were all significantly larger than 1 (corrected *P* < 0.01 under FS, SFi, and SFc conditions); thus time dilation occurred under these three conditions. Notably, under SFi or SFc conditions, the target itself was not flickering, so the time dilation could be attributed to the effects of the flickering distractor.

The estimated stimulus duration was larger under FS condition than under SFi or SFc conditions (corrected *P* < 0.01 in both comparisons). Therefore, the presence of a flickering target had a greater effect on time dilation than did a flickering distractor. SFi condition had a larger effect than SFc condition (corrected *P* < 0.05). The distractor state was the same under both of these conditions, as was the distance from the target in the visual field. The effect of the distractor, however, differed depending on whether it was displayed ipsilaterally or contralaterally to the target.

Time dilation occurred under FS condition, as has been found in previous studies. However, time dilation also occurred under SFi and SFc conditions, and to our knowledge this has never been reported in previous studies. Under these two conditions, the target stimulus to which the participants paid attention was stable. Nevertheless, the reproduced durations of the target stimulus were dilated because of the presence of the task-irrelevant flickering distractor.

The presence of flickering distractor induced time dilation, even when the target itself was stable. In addition, the effect of flickering distractor depended on the cortical distance: the flickering distractor ipsilateral to the target caused greater time dilation (SFi condition) than did the flickering distractor contralateral to the target (SFc condition)(Fig. 4a). These results are consistent with the hypothesis that time dilation is caused by neural entrainment^14^,^33^. Since previous studies have indicated that neural entrainment can be evoked by a task-irrelevant flickering stimulus and that neural entrainment can propagate^24^,^25^,^26^,^27^, the flickering distractor would have the effect of neural entrainment. Furthermore, A flickering distractor located ipsilateral to the target would have a stronger neural entrainment effect than one contralateral to the target, because the distance between the cortical areas responsible for each stimulus is smaller when the distractor is ipsilateral than when it is contralateral. Thus, the finding that the amount of time dilation was dependent on the cortical distance between the flickering distractor and the target is consistent with the idea that weaker entrainment causes weaker time dilation and that stronger entrainment causes stronger time dilation^14^.

### Experiment 2

In Experiment 1, the distractor duration was much longer than the target duration in order to maximize the effect of flickering distractors. In Experiment 2, we made the distractor duration almost the same as the target duration in order to prove the effect of the distractor duration and to determine whether a shorter distractor duration was sufficient to cause time dilation. Note that the distractor duration was jittered to ensure that the participants reproduced the target duration, not the distractor duration. For each participant, the correlation between the distractor duration and the reproduced duration was calculated for each target duration. Among 30 correlation coefficients (3 target durations by 10 participants), 18 were significant (*P* < 0.05), but they ranged from 0.14 to 0.31, indicating that the effects were rather weak. This meant that the participants performed the reproduction task on the basis of the duration of the target, not the distractor.

The estimated effects under FS, SFi, and SFc conditions are shown in Fig. 4b. These estimated values are the ratios of reproduced durations compared with that under SS condition. The ratios for FS, SFi, and SFc conditions were all significantly larger than 1 (corrected *P* < 0.01 under FS, SFi, and SFc conditions), indicating that time dilation occurred under all three conditions. Despite the fact that the distractor duration was almost the same as the target duration, time dilation of the target duration was still observed under SFi and SFc conditions, even though the target itself was stable under these conditions. This again confirmed the effect of the flickering distractor on the perception of target duration.

As in Experiment 1, the estimated value was larger under FS condition than under SFi or SFc conditions (corrected *P* < 0.01 in both comparisons). A flickering target therefore still had a larger time dilation effect than did a flickering distractor. However, the difference between SFi and SFc, which was significant in Experiment 1, was not significant in Experiment 2 (uncorrected *P* = 0.29).

We observed the time dilation induced by the flickering distractor whose duration was almost the same as the target. Again, this result suggests that the presence of flicker is critical for flicker-induced time dilation. Nevertheless, a flickering distractor located ipsilateral to the target induced as much time dilation as did one contralateral to the target; this was inconsistent with the results of Experiment 1. In Experiment 2, the distractor duration was much shorter than the distractor duration in Experiment 1. Because SSVEP, which originates from neural entrainment^28^, has the latency of a few hundreds of milliseconds from stimulus onset^18^, the length of flickering distractors in Experiment 2 might have been too short to affect the perception of the target.

## General Discussion

We demonstrated here that the presence of a task-irrelevant flickering distractor dilated the perceived duration of the target stimulus, which was spatially distant from the distractor. Furthermore, when the distractor duration was much longer than the target duration, the effect of flickering distractors depended on the cortical distance: distractors ipsilateral to the target induced larger time dilation than distractors contralateral to the target.

Several studies have argued that stimulus properties such as saliency, temporal frequency, or speed are critical factors in flicker-induced time dilation^8^,^12^,^16^. We found here, however, that flicker-induced time dilation was observed even when the target stimulus was stable. The duration of the stable target was perceived to increase when the task-irrelevant distractor was flickering. This result is consistent with the idea that the neural entrainment is critical for flicker-induced time dilation. It is true that the flickering distractors may have reduced the observer’s attention to the target. Nevertheless, generally, more attended events are perceived as longer and less attended events are perceived as shorter^34^,^35^,^36^. It is therefore unlikely that the effect of the flickering distractor was attributable to a reduction of attention to the target, because if that were the case then less attended target stimuli would be perceived as shorter.

In addition, the ipsilateral flickering distractor induced greater time dilation than the contralateral flickering distractor when the distractor duration was long enough. This result is also consistent with the prediction from the simulation of computational model based on the neural entrainment of oscillators^14^, which demonstrated that weaker neural entrainment induces smaller time dilation and stronger neural entrainment induces greater time dilation. Even though the ipsilateral distractor was as far from the target as the contralateral distractor in the visual field, the cortical areas responsible for ipsilateral distractor were closer to those for the target than were those for the contralateral distractor. Our results suggested that the extent of time dilation depends upon cortical distance, probably because the effects of neural entrainment on target perception are stronger when the cortical distance between the target and the distractor is smaller. It should to be noted that we only assumed that the visual flickers cause neural entrainment on the basis of previous studies that observed robust SSVEP with flickering visual stimuli^17^,^18^,^19^,^20^,^21^,^22^,^24^,^25^,^26^,^27^,^28^. Studies using EEG or Magnetoencephalogram (MEG) measurements are needed to further assess the role of cortical distance in the time dilation. Recently, by using EEG, Horr, Wimber, and Di Luca^33^ measured pairwise phase consistency (PPC) as another index of neural entrainment. They used an auditory click to induce time dilation and demonstrated the correlation between the magnitude of PPC and the amount of dilation. Although this time dilation was induced not by visual flicker but by an auditory click, their result also suggests that flicker-induced time dilation is caused by neural entrainment.

It is possible that the ipsilateral flickering distractor reduced attention to the target more than did the contralateral flickering distractor, and that this difference in attention induced different amounts of time dilation. However, this is unlikely to be the case, because it has been reported that higher-level attention does not differ between conditions in which attention is divided across the hemifield and within the hemifield^22^. Moreover, although less-attended stimuli should be perceived as shorter, as described above, the ipsilateral flickering distractor elongated the perceived target duration. It is also possible that flickering distractors increased the general arousal level and elongate the perceived duration. New and Scholl^32^ found that oddballs spatially separated from the target elicit subjective time expansion regardless of their distance from the target, and concluded that the general arousal level increased by oddballs influence subjective time dilation as a global visual experience. However, our results differ from the above findings in that the extent of time dilation depended on the cortical distance between the flickering distractor and the target. This suggests that flicker-induced time dilation has a different mechanism other than attentional modulation or general arousal level.

Droit-Volet and Wearden^5^ have demonstrated time dilation using a visual flicker presented before the timed stimulus and attributed the effect to modulations of an internal clock’s speed. Although they have not argued for the neural implementation of an internal clock, their results are consistent with ours in suggesting that visual flickers modulate some sort of clock mechanism. Our study further illustrates that a visual flicker spatially distant from the target can also induce time dilation, and that the effect of this modulation depends on the cortical distance.

There are several models for time perception. Ivry and Schlerf^37^ classified them into two fundamental frameworks: dedicated models and intrinsic models. While dedicated models assume the presence of specialized neural mechanisms for the representation of temporal information, intrinsic models assume that temporal information is inherent in neural dynamics and that there is no specialized neural mechanism for representing this information. In the case of state-dependent models, which are intrinsic models, temporal processing emerges from the internal dynamic state of the neural networks, which change activity patterns depending on the interval from the event onset^38^,^39^. It is possible that the flickering distractor alters the activity pattern of the neural network, which would then result in the distortion of the perceived duration. However, based on the framework of this model, it is hard to explain why the flickering distractors have different dilation effects depending on their cortical distance to the target.

Another intrinsic model, the energy readout model, assumes that duration information is encoded in the magnitude of neural activity. This model can predict the subjective time expansion due to attentional capture^36^,^40^. Since paying attention enhances neural activity^41^,^42^, attended events would be perceived as longer. As discussed above, it seems difficult to explain our results based on this model, as the flickering distractor would reduce attention to the target and thus lead to time compression. However, it is possible that the flickering ipsilateral distractor increases the activity of early visual cortical neurons responsible for the target stimulus, since some neurons in the early visual cortex have receptive fields that cross the horizontal meridian^43^,^44^. On the other hand, the flickering contralateral distractor would not be included in the receptive fields of early visual cortical neurons responsible for the target. Based on the energy readout model and the receptive field characteristics of the early visual cortex, the activity magnitudes of the activities of early visual cortical neurons responsible for the target may explain our results: large activities would be induced when the target itself is flickering, whereas medium and smaller activities would be induced by the flickering ipsilateral distractor and the flickering contralateral distractor, respectively. The supposed order of activity magnitude is consistent with the results of Experiment 1. Nevertheless, the interpretations based solely on the magnitude of the activity of the early visual cortex cannot explain why the flickering contralateral distractor induces time dilation.

The pacemaker-accumulator model is one of the dedicated models. This model assumes that the pacemaker and the accumulator encode duration information^45^,^46^. The pacemaker generates the rhythmic pulses and the accumulator counts the pulses during the interval. Duration information can then be readout from the number of counted pulses. Thus, in this model, the perceived duration would increase with increases in the pulse rate generated by the pacemaker, and flickering stimuli with higher temporal frequencies would induce larger time dilations. Since only one temporal frequency was used in our experiments, our results do not exclude the possibility that the pacemaker-counter model explains flicker-induced time dilation. That explanation is likely to be implausible, however, as several previous studies have shown that the extent of dilation caused by the flicker does not increase with increases in the flicker’s temporal frequency^12^,^14^.

The Striatal-Beat Frequency (SBF) model is another example of a dedicated model. In the SBF model, which was proposed on the basis of neurophysiological experiments on rats, duration information is encoded by the oscillator and detector components^47^,^48^. This model assumes that the detector is projected by a number of oscillator neurons in the cortex and that the detector can distinguish the patterns of neural activity in the oscillators, which change depending on the duration. While it is difficult to apply this neural model to human perception, it suggests the possibility that the distortion of the perceived duration can be explained by the modulation of neural oscillators. In fact, Hashimoto and Yotsumoto^14^ simulated flicker-induced time dilation in humans by incorporating neural entrainments in the existing SBF model. Results of the present study suggest that neural entrainment is an important factor for time dilation, and that their effects are dependent on the cortical distances. Thus, the results of our study may be able to be discussed in the framework of the SBF model.

In conclusion, here we demonstrated the spatial distribution of the flicker effect in time dilation. First, time dilation can be induced by flickering stimuli spatially remote from the target to be timed. Second, the flickering ipsilateral distractor induced larger time dilation than the flickering contralateral distractor, when the distractor duration was long enough. These findings suggest that the presence of flicker induces time dilation depending on the cortical distance between the flicker and the target. This is consistent with the recent study reporting that neural entrainment is critical for flicker-induced time dilation.

## Methods

### Participants

In Experiment 1, ten naïve volunteers and one author (MO) participated (8 male; age range: 19–24 years). All were right handed. In Experiment 2, nine naïve volunteers and one author (MO) participated (10 male; age range: 19–23 years). One participant was left handed. All participants had normal or corrected-to-normal vision. All participants gave written informed consent to participate in the experiment in accordance with the Declaration of Helsinki. The protocol was approved by the institutional review boards of the University of Tokyo, and all experiments were carried out in accordance with the guidelines set by the Ethics Committee of the University of Tokyo.

### Apparatus

The experiment was conducted in a darkened room. Participants sat in front of a 23-inch LCD monitor at a distance of 57.3 cm, with their heads on a chin rest. The monitor was a screen unit of Tobii TX300 Eye Tracker (Tobii Technology AB, Stockholm, Sweden) with 1920 × 1080 pixel resolution at a refresh rate of 60 Hz. The actual temporal frequencies of the flickering stimuli were measured using a photodiode and an oscilloscope. The frequency spectrum of the flickering stimuli ranged from 9.5 Hz to 10.5 Hz with a sharp peak at 10 Hz. The Eye Tracker unit (300-Hz sampling rate) was used for eye tracking. The experiment was conducted with Matlab 2014b (The MathWorks Inc., Natick, MA, USA) using Psychophysics Toolbox extensions^49^,^50^,^51^ and a Tobii Analytics Software Development Kit.

### Stimuli

**Figure 1:**
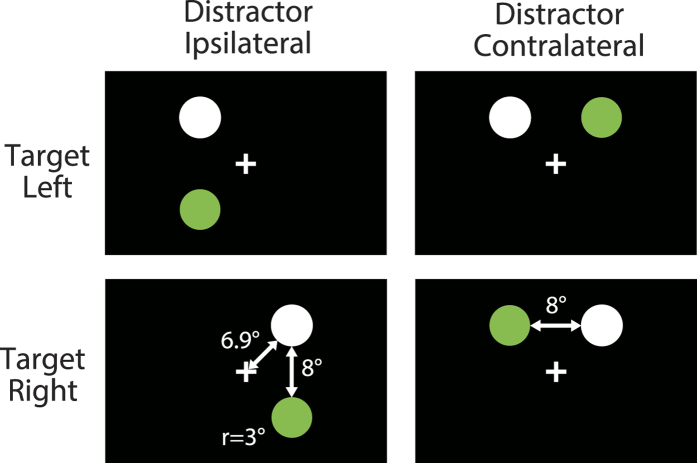
Stimulus configurations. The target was the white disk and appeared in the upper right or upper left of the display. The distractor was a green disk. The ipsilateral distractor appeared in the same visual hemifield as the target, and the contralateral distractor appeared in a different visual hemifield. The distance between target and distractor was constant regardless of whether the distractor was ipsilateral or contralateral.

A white fixation cross was presented in the center of the display against a black background (0.3 cd/m^2^). Two stimuli—target and distractor—were used in the experiment (Fig. 1). The target stimulus was a white disk (108 cd/m^2^) with a radius of 3°; its center was 7° left or right of, and 7° above, the fixation cross. As the distractor stimulus, a green disk equiluminant to the target and with a radius of 3° was displayed either ipsilateral or contralateral to the target. The center of the ipsilateral distractor was positioned 14° below the center of the target. The center of the contralateral distractor was positioned 14° to the right of the left target or 14° to the left of the right target. The distance between target and distractor was 8° from edge to edge; this distance was constant regardless of whether the distractor was ipsilateral or contralateral. The stimuli were either stable or flickering at 10 Hz.

The spatial configurations of the stimuli were the same in the two experiments, but the time course was different. In Experiment 1, the distractor duration was much longer than the target duration (Fig. 3a).

In Experiment 2, the distractor duration was almost the same as the target duration (Fig. 3b). To ensure that the participants reproduced the duration of the target, not the distractor, the distractor duration was jittered. The stimulus onset asynchrony between the distractor and the target was −30% to +30% of the target duration (where a negative value indicates earlier distractor onset than target onset, and a positive value indicates the opposite sequence). The stimulus offset asynchrony between distractor and target was also −30% to +30% of the target duration.

### Procedure

In both experiments, we employed the reproduction task, which is used in several previous studies about interval time perception^8^,^16^,^52^. The participants were instructed to reproduce the duration of the target stimulus, while ignoring the distractor stimulus.

In Experiment 1, the distractor appeared at the beginning of the trial, and then the target appeared 2000 to 2500 ms after the onset of the distractor (Fig. 3a). The distractor remained during and after target presentation. The target duration was 450, 650, or 850 ms. The distractor disappeared 1000 to 1500 ms after target offset. The fixation cross then turned red and participants were required to reproduce the target duration by pressing the space key on the keyboard. When the space key was released, the trial ended. The ITI was 250 to 500 ms.

In Experiment 2, the stimuli appeared 500 to 1000 ms after the onset of the trial (Fig. 3b). The target duration was 450, 650, or 850 ms. The distractor was displayed in accordance with the time course described above. After the disappearance of the target and distractor stimuli, the fixation cross turned red and the participants were required to reproduce the target duration by pressing the space key on the keyboard. When the space key was released, the trial ended. The ITI was set to 250 to 500 ms.

**Figure 2:**
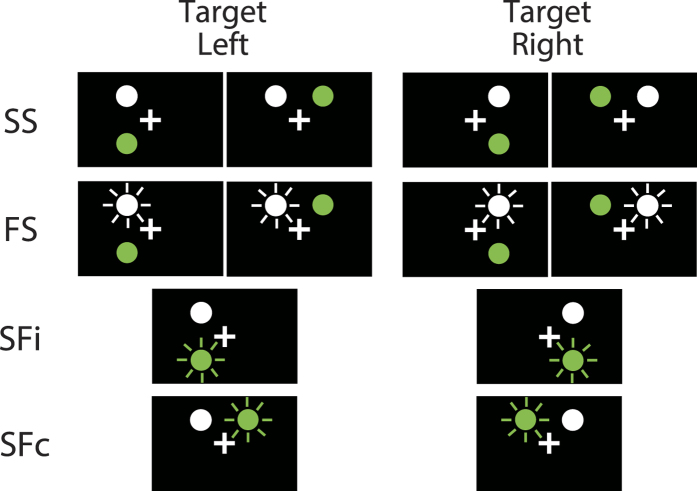
Schematics of the experimental conditions. SS condition: target stable, distractor stable. FS condition: target flickering, distractor stable. SFi condition: target stable, distractor flickering in the visual field ipsilateral to the target. SFc condition: target stable, distractor flickering in the visual field contralateral to the target.

**Figure 3:**
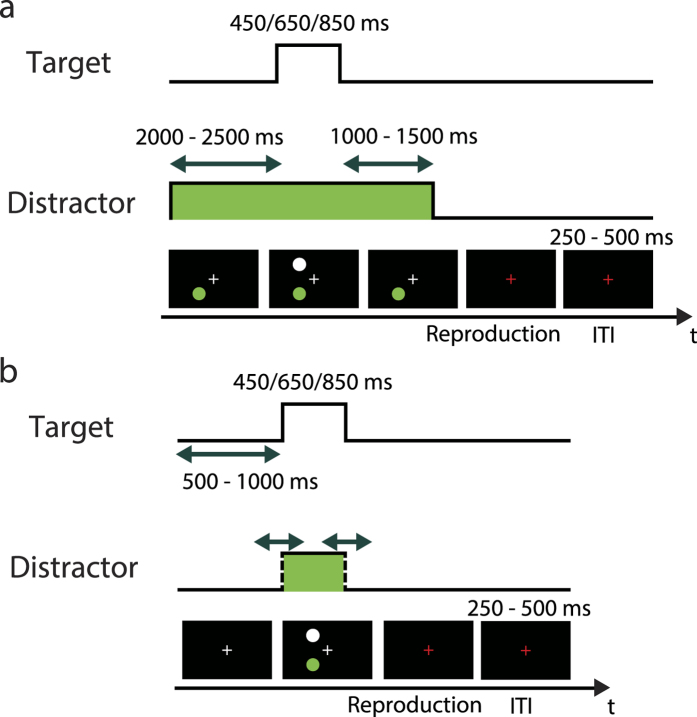
Time course of a trial. (**a**) In Experiment 1, the distractor duration was much longer than the target duration. After presentation of the stimuli, the fixation cross turned red and participants were required to reproduce the target duration by pressing a button. No feedback was provided on the reproduction performance. The trial ended when the button was released. After the intertrial interval (ITI), the next trial started. (**b**) In Experiment 2, the distractor duration was almost the same as the target duration. To prevent participants from reproducing the distractor duration, the distractor onset and offset were jittered. The stimulus onset asynchrony between the distractor and the target was −30% to +30% of the target duration (where a negative value indicates an earlier distractor onset than target onset, and a positive value indicates the opposite sequence). The stimulus offset asynchrony between the distractor and the target was also −30% to +30% of the target duration.

**Figure 4:**
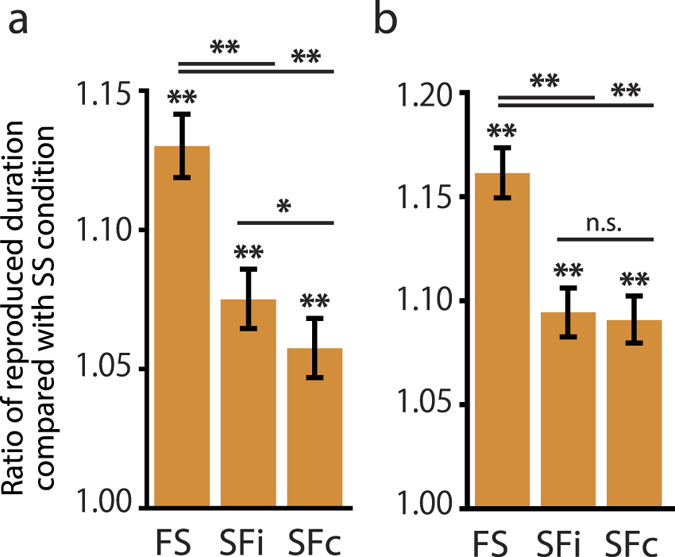
Estimated ratio of reproduced duration under each condition compared with that under SS condition. (**a**) In Experiment 1, the ratio was significantly larger than 1 under FS, SFi, and SFc conditions. The differences between FS and SFi, FS and SFc, and SFi and SFc were also significant. (**b**) In Experiment 2, the ratio was still significantly larger than 1 under FS, SFi, and SFc conditions. The differences between FS and SFi and between FS and SFc were also significant, although there were no significant difference between SFi and SFc. *P < 0.05; **P < 0.01. Error bars indicate 95% credible intervals.

Each experiment included four conditions that were named on the basis of whether the target and the distractor were stable or flickering and whether the distractor was ipsilateral or contralateral (Fig. 2). Under SS condition (Stable target and Stable distractor), a stable target and stable distractor were displayed. Because no differences in effect were observed between the ipsilateral and contralateral stable distractors, in the preliminary experiment we merged these two into the SS condition. In one-fourth of the trials under SS condition, the target was presented in the left visual field and the distractor was ipsilateral to the target, whereas in another one-fourth the target was presented in the left visual field and the distractor was contralateral to the target. In the other half of the trials under SS condition, the target was presented in the right visual field and the distractor was ipsilateral or contralateral. Under FS condition (Flickering target and Stable distractor), a flickering target and a stable distractor were displayed. Again, on the basis of our preliminary experiments, both ipsilateral and contralateral stable distractors were included under FS condition. As with the SS condition, the right or left target and the ipsilateral or contralateral distractor were each shown in one-fourth of the trials under FS condition. Under SFi condition (Stable target and Flickering distractor ipsilateral), a stable target and a flickering distractor ipsilateral to the target were shown. In half of the trials under SFi condition the target was presented in the right visual field, whereas in the other half it was presented in the left. Under SFc condition (Stable target and Flickering distractor contralateral), a stable target and flickering distractor contralateral to the target were shown. In half of the trials under SFc condition the target was presented in the right visual field, whereas in the other half it was presented in the left.

Each experiment consisted of four sessions. Within each session the target position was constant, so that participants knew where the target was going to appear. The sequence of the target was counterbalanced across participants (Left – Right – Right – Left target, or Right – Left – Left – Right target). In one session, each condition was displayed 15 times for each target duration, resulting in a total of 180 trials per session. Each condition had 60 trials for each target duration after the right and left targets had been merged.

### Analysis

After the analysis of eye-tracking data, trials in which participants had moved their eyes more than 2° from the fixation point were excluded from further analysis.

To dissociate the fixed effect of time dilation from the random effect of the individual, we applied Bayesian inference to our statistical analyses. The target positions (right or left) were broken down into one condition. The reproduced durations for each condition and target duration were fitted into a normal distribution. We modeled each of the mean and the standard deviation as the product of multiplication of the fixed effects of condition and the sum of the fixed effects of target duration and the random effects of individuals. We repeatedly generated 2000 samples by applying Markov-chain-Monte-Carlo sampling, and we used the latter 1000 samples for analysis. By performing the sampling three times, we estimated the fixed effects of condition and target duration as the 50th-percentile points of 3000 samples. Markov-chain-Monte-Carlo sampling was conducted by using R^53^ and RStan^54^.

## Additional Information

**How to cite this article**: Okajima, M. and Yotsumoto, Y. Flickering task–irrelevant distractors induce dilation of target duration depending upon cortical distance. *Sci. Rep.* **6**, 32432; doi: 10.1038/srep32432 (2016).
